# A “Rod” in Pickle for the Bacillus Tuberculosis

**Published:** 1882-10

**Authors:** 


### Editorial.

---

### A "Rod" in Pickle for the Bacillus Tuberculosis.

We propose to publish at no distant date a contribution to the literature of a subject which, though it concerns a very minute body, has been lately gaining in magnitude with every day. Those who read these pages need scarcely be told that, under the persuasive influence of various aniline stainings, bacteria are now daily projected into view in sections of tissues of the living body affected with a no small number of diseases. Koch's discovery of the bacillus of tuberculosis has in this way attracted an attention of the profession and the public which probably exceeds that directed to any similar subject during the past year.

Our readers will remember the position taken by Dr. H. D. Schmidt, of New Orleans, with reference to the bacillus lepræ, in his admirable paper upon that subject which lately appeared in this journal. They who were then arrested by the evident candor of the author, his unquestioned desire to arrive at nothing but exact truth, and his actual submission of the fruits of his labor to the critical examination of a special committee, will anticipate with no little pleasure the perusal of a paper upon an allied subject from the same master hand.

The essay to which we now refer, and which will embody the results of the late studies of the bacilli of tuberculosis conducted by Dr. Schmidt, is based upon observations begun by him during the fall and winter of 1881-1882. These extensive microscopical researches concerned originally the structure. evolution and involution of miliary tubercle. The announcement by Koch of his discovery of the bacillus of tubercle, was made during the progress of this investigation, and naturally served to stimulate Dr. Schmidt to an extensive series of experiments in connection with this subject. He at once prepared a number of specimens of sputa and thin sections of tuberculous lung tissue, which were stained according to the directions of Koch, as originally given in the *Berliner Klinische Wochenschrift.* But though these staining fluids, methyl-violet and vesuvin, were prepared with the greatest precautions and in exact accordance with Koch's instructions, bacilli by their aid could not be detected. In some specimens of sputa, it is true, certain bodies became visible, having a faint bluish color, which were presented in the form, and of about the size, of small bacilli. Eventually the same phenomena were discovered in thin sections taken from other cases of the same disease. Failing thus to detect the bacillus in sections stained by Koch's own method, Dr. Schmidt had recourse to the old and well-tried bacteria detective, a solution of caustic potash in the strength of thirty per cent. By this method, the presence was established in tubercular tissue of minute rods, which in every respect corresponded to the description given of Koch's bacilli. The same results were obtained after the examination of a number of specimens of sputa. It was noticed, however, that in the examination of the first sections with caustic potash, these rods were almost always found in the company of a group of larger and smaller fat globules; and the idea was naturally suggested to the mind of the distinguished microscopist, that these rods, being very highly refractive, might represent minute crystals of fat. It will be remembered that he had observed groups of minute crystals of margaric acid in the degenerating fat cells seen in leprous tissue.

This idea grew to be more than a suspicion, in consequence of the fact that during the course of the last summer, he had made a large number of sections of the different organs involved in four fatal cases of yellow fever. Some of these, obtained from the liver, heart and kidney, were one day treated with the solution of caustic potash, with a view to determine the presence or absence of bacteria. In consequence of the fatty degeneration which the organs in these cases had undergone, the tissues under examination contained an unusual quantity of fat. When examined under the microscope, bacteria were not detected in any of the slides, and the latter were thus laid aside upon the table for further examination. Upon the next day, Dr. Schmidt chanced to put one of these again under his objectives, when he discovered, to his astonishment, that it was covered with the most beautiful fat crystals. The same was true of the two other sections, but in all, the figures formed by the minute needles exhibited a difference, showing that the crystallization had taken place under the influence of the tissues themselves. Similar results were obtained by treating tubercular sputa with the potash solution.

We shall occupy no further space in giving details respecting the instructive and fertile theme which is thus suggested. In the paper which we shall publish at no distant date, they who are interested in this subject can follow with our author the line of observation which he has pursued in his remarkable experiments. Let it suffice to add here, that we can confidently assert that every form of Koch's bacillus of tubercle can be artificially produced in all its details by Dr. Schmidt, in every tissue that contains fat.

At a date subsequent to the publication of Koch's method of staining, that of Ehrlich, and also that of Gibbes, were placed before the public. Procuring the aniline oil and the chrysoidine from New York, Dr. Schmidt employed these also with the greatest precision and precautions, obtaining always the same results, that is, the discovery of the rods, but not in a stained condition. In tuberculous tissue, these rods are by no means found to correspond with each other in the matter of form and size. They are generally recognizable in those elements of the tubercular mass which have undergone fatty degeneration, but always in the company of fat globules, and frequently, also, of pigment granules. They are best recognized after treatment with the potash solution, because the latter renders clear the protoplasm of the cells, but eventually destroys it.

At this date Dr. Schmidt has made and examined, by various methods, hundreds of different sections, taken from more than a dozen different cases of phthisis, as also numerous specimens of sputa. The rods correspond in every particular with the descriptions given by Koch of his bacillus, though as yet they have not been stained. They are highly refractive, and in both stained and unstained sections have a faint bluish color, which has led Schmidt to suspect that they have been supposed by other observers to have actually undergone staining when they had not. The "rods," or, as we shall hereafter call them, crystals, contained within the cells, have been generally found at the margin of the tubercle; very frequently, however, they have been recognized among groups of pigment granules; in some cases, even, large prismatic fatty crystals have been discovered, which, upon closer examination, proved to be composed of minute rods or needles. Sometimes the crystals occurred in the form of plates. All of these forms, however, observed in tuberculous tissue, have been artificially produced in sections which were non-tuberculous.

Let it be distinctly understood, that the position assumed by the distinguished pathologist of New Orleans is not as to the bacilli which Koch himself has seen, though we understand that some of Koch's own preparations are at this moment *en route* to Schmidt's laboratory, where they will be compared with those fat crystals, so-called bacilli, which our author has found to be so marvelously like the bacteria of German importation.

We do not say that the bacillus epidemic, if it may be so termed, will happily terminate in a fat globule and a rod-like crystal of margaric acid. *Parturiunt montes; nascitur ridiculus mus!* What we do say is, that such an issue, if it conclude the controversy, will be one for which we shall all be indebted to the patient, modest, and untiring devotion of the pathologist of the Charity Hospital in New Orleans. Whichever horn of the dilemma our readers may conclude to take, our part is done in giving them the assurance that there is a "rod" in pickle for the bacillus of tubercle, and that it is to be found not far from the delta of the Mississippi!

---

A VALUABLE sign of death is the lack of muscular contraction under an electric current, two or three hours after the heart has ceased to beat.

